# Editorial: Nutritional Aspects of Immunity and Immunometabolism in Health and Disease

**DOI:** 10.3389/fimmu.2020.595115

**Published:** 2020-10-07

**Authors:** Erik A. Karlsson, Melinda A. Beck, Nancie J. MacIver

**Affiliations:** ^1^Virology Unit, Institut Pasteur du Cambodge, Phnom Penh, Cambodia; ^2^Department of Nutrition, Gillings School of Global Public Health, University of North Carolina, Chapel Hill, NC, United States; ^3^Department of Pediatrics, Duke University Medical Center, Durham, NC, United States; ^4^Department of Immunology, Duke University Medical Center, Durham, NC, United States; ^5^Department of Pharmacology and Cancer Biology, Duke University Medical Center, Durham, NC, United States

**Keywords:** nutrition, immunology and infectious diseases, obesity, undernutrition, micronutrient deficiency, immune metabolism, micobiome, influenza

In his 1825 work Physiologie du Gout, ou Meditations de Gastronomie Transcendante, French lawyer and famous gastronome, Jean Anthelme Brillat-Savarin wrote “Dis-moi ce que tu manges, je te dirai ce que tu es.” [Tell me what you eat: I will tell you what you are]. Later, in the mid-1860s, German philosopher Ludwig Andreas Feuerbach wrote “Der Mensch ist, was er ißt.” [Man is what he eats]. However, it wasn't until the 1920s that nutritionist Victor Lindlahr, the father of the Catabolic Diet, wrote (in an advertisement for beef, no less) “Ninety per cent of the diseases known to man are caused by cheap foodstuffs. You are what you eat.”

You are what you eat. It may just seem like a ubiquitous aphorism, but within its simplicity lies a salient truth. Our bodies contain the nutrients that we eat, and all of our physiological functions and systems are based on those nutrients and their availability. Overall, our immune system is highly impacted by food consumption and utilization. Indeed, it is increasingly becoming apparent that immune cell number and function are altered in response to changes in nutritional status.

Alteration in the nutritional status of an organism can affect its immune system in a variety of ways. With the world facing a “triple burden” of malnutrition amidst the global health crisis that is the novel coronavirus disease 2019 (COVID-19) pandemic, this Research Topic was envisioned to contain a collection of review articles which touch on several aspects important in the field of nutritional immunology.

Universally, under- and overnutrition are major factors in health and disease. Approximately 45% of deaths in children under the age of 5 can be linked to undernutrition while 1 out of every 4 people on the globe are overweight. Undernutrition impairs immune cell survival, proliferation, and function, leading to poor response to infection or vaccination. Conversely, obesity leads to the establishment of a low-grade chronic inflammatory state with an increase in pro-inflammatory cells and circulating pro-inflammatory cytokines, resulting in altered cellular immunity and increased rates of certain forms of autoimmunity. To this end, Bourke et al. discuss the impact of childhood undernutrition on immune cell dysfunction and Liu and Nikolajczyk discuss how tissue resident immune cells can fuel obesity-associated inflammation linked to poor immune response. Further, Horwood et al. describe how changes to a Western diet in the Pacific region have led to obesity and possible links with disease severity, and Honce and Schultz-Cherry describe a large body of work done correlating obesity to the pathogenesis of influenza.

Aside from macronutrient imbalance, micronutrient deficiency, sometimes termed “hidden hunger,” also represents a major global health issue and contributing factor in altered nutritional immunology. Vitamin deficiencies, especially Vitamin A, affect more than half of all countries in the world, especially in low-income countries in Africa and South-East Asia. Young children and pregnant women are particularly susceptible to the effects of these deficiencies. Penkert et al. discuss the impact of vitamin A deficiency on a very important topic, vaccine immunogenicity and effectiveness.

Mechanistically, one of the key determinants for nutritionally-regulated immune dysfunction is by altering the systemic metabolic state, which can then influence immune cell metabolism. Thus, understanding the link between systemic metabolism and immune cell metabolism is important as the changes in immune cell metabolism can directly lead to an altered immune response. Ieronymaki et al. review how insulin signaling and insulin resistance in macrophages can lead to impaired trained immune responses. Both Honce and Schultz-Cherry and Liu and Nikolajczyk discuss how metabolic changes in obesity could also affect immune responsiveness in the obese host.

There is also an emerging interest in understanding the role of the microbiome (both bacterial and viral) in the context of immune cell response to changes in nutrition. Indeed, it is now clear that dysbiosis can influence both systemic metabolism and immunity, and vice versa. Horwood et al. discuss how gut microbiome differences and changes in the Pacific vs. the Global North could impact disease resistance and severity. Further, Leshem et al. discuss the potential immunodulatory and therapeutic effects of fecal microbiota transfer.

Overall, the papers contained in this Research Topic present a good review of knowledge which could have significant impacts on the current worldwide pandemic. COVID-19 represents a major issue in some of the most under- and overweight populations in the world. While the impact of underweight is yet understudied, more and more data is emerging that obesity is a major factor in COVID-19 morbidity and mortality. In addition, the global race for a vaccine represents a major concern for nutritionally compromised populations, especially since the majority of seroconversion and effectiveness studies are performed on healthy individuals.

Aside from the current nutritional status of the global population, times of plague have long been associated with times of famine. The COVID-19 pandemic has already caused global economic reduction, disruption of food production and transportation systems, and delays and destruction of vital aid programs, presenting a future where more and more individuals are nutritionally compromised. These issues will not only lead to greater morbidity and mortality from the COVID-19 pandemic, but will also perpetuate the vicious cycle of malnutrition and infectious disease.

Therefore, the scientific literature reviewed here is a vital collection of understanding the impact of nutritional status on the immune system and immunometabolism. Further insights into the role of specific nutrients, nutritional states, and neutraceuticals are crucially important, not only for maintaining health, but also for fighting against endemic and emerging infectious diseases.

## Author Contributions

All authors listed have made a substantial, direct and intellectual contribution to the work, and approved it for publication.

## Conflict of Interest

The authors declare that the research was conducted in the absence of any commercial or financial relationships that could be construed as a potential conflict of interest.

